# Influence of a 3′ Terminal Ribozyme on AgoshRNA Biogenesis and Activity

**DOI:** 10.1016/j.omtn.2019.04.001

**Published:** 2019-04-08

**Authors:** Elena Herrera-Carrillo, Zongliang Gao, Ben Berkhout

**Affiliations:** 1 Laboratory of Experimental Virology, Department of Medical Microbiology, Amsterdam UMC, University of Amsterdam, Meibergdreef 15, 1105 AZ Amsterdam, the Netherlands

**Keywords:** HIV, RNAi, Dicer, Ago2, ribozyme, Dicer-independent shRNA, miR-451

## Abstract

Short hairpin RNAs (shRNAs) can induce gene silencing via the RNA interference (RNAi) mechanism. We designed an alternative shRNA molecule with a relatively short base-paired stem that bypasses Dicer and instead is processed by the Argonaute 2 (Ago2) protein into a single guide RNA strand that effectively induces RNAi. We called these molecules AgoshRNAs. Active anti-HIV AgoshRNAs were developed**,** but their RNAi activity was generally reduced compared with the matching shRNAs. In an attempt to further optimize the AgoshRNA design, we inserted several self-cleaving ribozymes at the 3′ terminus of the transcribed AgoshRNA and evaluated the impact on AgoshRNA processing and activity. The hepatitis delta virus (HDV) ribozyme is efficiently removed from the transcribed AgoshRNAs and generates a uniform 3′ overhang, which translates into the enhanced antiviral activity of these molecules.

## Introduction

Since the discovery of the RNA interference (RNAi) process in *Caenorhabditis elegans* in 1998, RNAi has become an important tool for selective silencing of the expression of target genes in a wide range of mammalian cells.^1^, ^2^, ^3^ RNAi can be induced by small interfering RNAs (siRNAs) that target complementary mRNAs for degradation or by plasmid-based vectors that express short hairpin RNAs (shRNAs) that are processed intracellularly into siRNAs.^4^, ^5^, ^6^, ^7^ siRNAs are small RNA duplexes of approximately 21 nucelotides (nt) long with a 2 nt overhang at the 3′ end. Man-made shRNAs with a stem of 20–29 base pairs (bp) and a loop of at least 5 nt are transcribed in the nucleus, transported to the cytoplasm by Exportin-5 and processed by Dicer into the active siRNA duplex. The siRNA duplex binds to Argonaute 2 (Ago2) and forms the RNA-induced silencing complex (RISC). The guide strand and the passenger strand are subsequently unwound, and the guide strand is exclusively retained, whereas the passenger strand is degraded or removed from the RISC. Which strand is incorporated as the guide into the RISC is mainly determined by thermodynamic properties of the duplex that are probed by Dicer.^8^, ^9^, ^10^ The guide strand, designed to be perfectly complementarity to the target mRNA, will subsequently induce mRNA degradation.^11^, ^12^, ^13^

Natural microRNAs (miRNAs) are also processed by Dicer but, alternatively, RISC can also accommodate certain pre-miRNAs in the absence of Dicer. More specifically, biogenesis of the unusually small miR-451 (17 bp stem and 4 nt loop), a miRNA present during erythrocyte differentiation, does not require Dicer.^14^, ^15^ miR-451 is instead processed by Ago2, which cleaves the duplex in the 3′ strand between bp 10 and 11, thus generating a single 30 nt guide strand that is further trimmed by poly(A)-specific ribonuclease (PARN) to create the ∼22−26 nt mature miR-451.^14^, ^16^, ^17^, ^18^ Recent studies have indicated that relatively short shRNAs (<19 bp) are also processed by Ago2 instead of Dicer.^19^, ^20^, ^21^, ^22^, ^23^, ^24^, ^25^ We called these molecules AgoshRNA, as both their processing and silencing function are mediated by Ago2, but other names have been coined in the literature (sshRNA, agsiRNA, agshRNA, and saiRNA).^24^, ^25^, ^26^, ^27^, ^28^ The AgoshRNA design has a clear advantage over regular shRNAs, in that no passenger strand is produced that may cause off-target effects. We previously listed other advantages, including the ability of AgoshRNAs to remain fully active in Dicer-negative cells such as monocytes.^29^

We previously converted potent anti-HIV shRNAs into AgoshRNAs by shifting the guide sequence from the 3′ to the 5′ side of a shortened hairpin, but this conversion affected the gene-silencing efficacy.^30^ In a subsequent attempt to optimize the AgoshRNA design, we extended the guide strand “over the loop,” but no increased knockdown potency was measured.^30^ The insertion of the evolutionary conserved miR-451 loop (AGUU) or the particularly stable CUUG tetraloop in AgoshRNA molecules also did not result in enhanced silencing activity.^31^ The insertion of a weak G-U bp at the top of the hairpin stem improved the silencing activity of AgoshRNA for some molecules, but the effect was not general.^19^ However, the introduction of a bottom mismatch and 5′ terminal A or G enhanced the AgoshRNA activity.^32^ As the commonly used RNA polymerase III (Pol III) promoters for small RNA expression prefer to start with a pyrimidine (G/A), expression of the 5′ A/G variants was found to be increased.^33^, ^34^ We selected the 5′ A over G because the middle (MID) domain of the human Ago2 protein prefers to load small RNAs with U or A as the 5′ end.^35^, ^36^ The following parameters for the design of optimized AgoshRNA molecules were defined: a small 5 nt loop and a duplex length of 18 bp with a bottom mismatch, and A is recommended as 5′ terminal nt when a Pol III promoter is used.^32^

The new design rules were used to create optimized AgoshRNAs against HIV, targeting either the cellular mRNA encoding the CCR5 receptor or the viral RNA.^37^, ^38^ We successfully designed AgoshRNAs that potently downregulated CCR5 expression on human T cells and peripheral blood mononuclear cells (PBMCs), without adverse effects on T cell development.^38^ CCR5 knockdown significantly protected T cells from infection by CCR5-tropic HIV strains. Previously validated anti-HIV shRNAs could also be converted into AgoshRNAs.^39^ More important, we demonstrated that a toxic shRNA can be converted into a non-toxic AgoshRNA, likely because no passenger strand is generated by the latter.^37^ Furthermore, AgoshRNA-based antivirals, unlike shRNA-based inhibitors, remained active in Dicer-minus monocytic cells that are host cells for HIV infection.^37^ These combined results suggest that the future for AgoshRNA therapeutics may be promising, but their RNAi activity was reduced compared with that of the matching shRNAs.

In this study, we attempted to generate AgoshRNA molecules with a more precise 3′ end by insertion of a self-cleaving ribozyme immediately downstream of the transcribed AgoshRNA. Pol III transcripts are usually terminated at a heterogeneous position within the regular T-stretch transcription termination signal (T6), thus creating RNAs with a variable U-tail of 1–6 nt.^40^ Although the length of the 3′ overhang of chemically synthesized AgoshRNAs has no impact on its association with Ago2 and its silencing activity in the 1–3 nt range, larger overhangs negatively influence the silencing activity because of impaired Ago2 binding.^24^ We hypothesized that insertion of a 3′ terminal ribozyme would generate a more discrete 3′ overhang in the context of the AgoshRNA duplex that might enhance the silencing activity. We evaluated the impact of this 3′ end modification on AgoshRNA processing and activity.

## Results

### Design of Anti-HIV AgoshRNA Molecules

**Figure 1:**
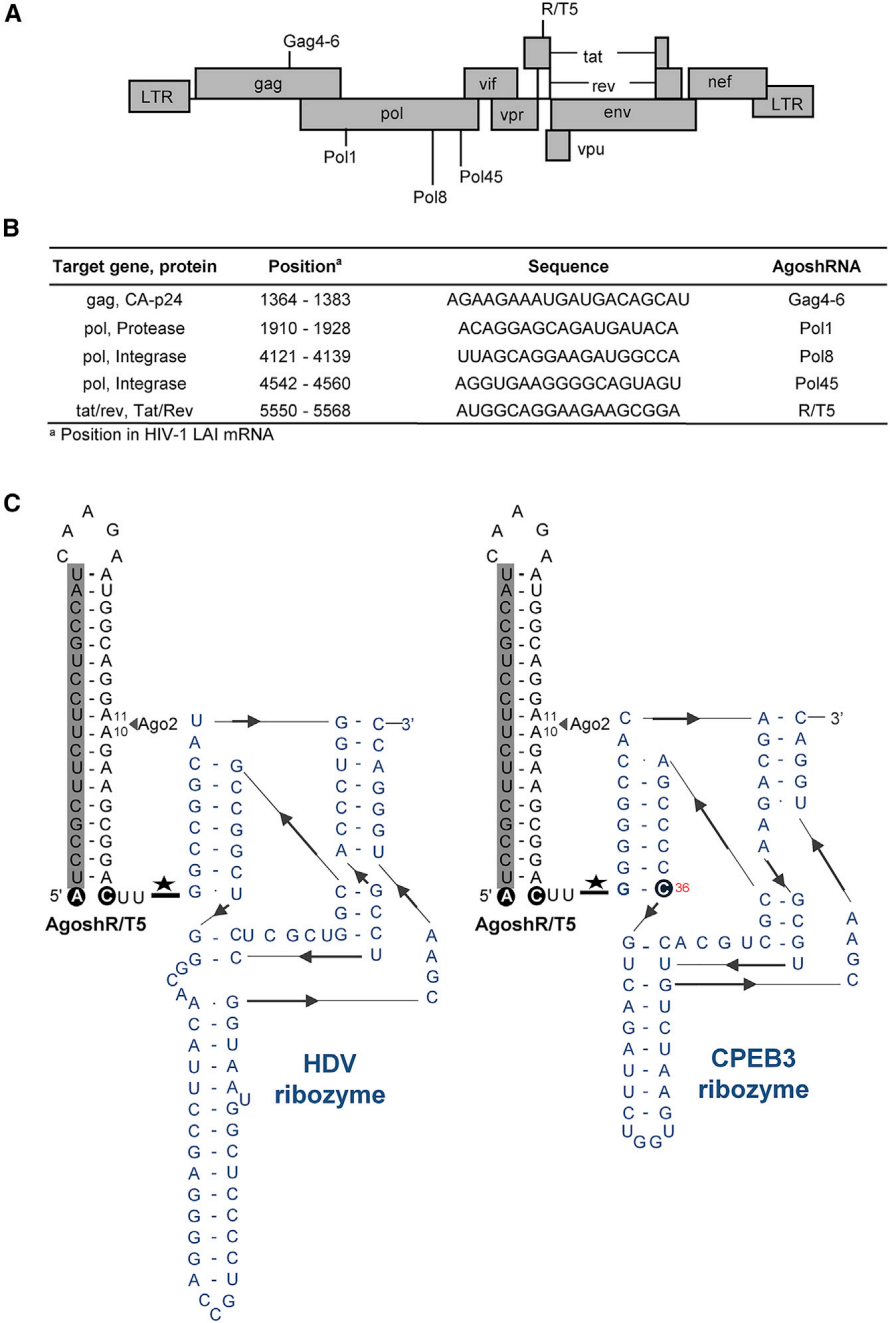
Design of Anti-HIV AgoshRNAs (A) The HIV genome showing the position of all target sites. (B) HIV target sequences that are highly conserved among HIV isolates. (C) Secondary structure of the R/T5 AgoshRNA with a 3′ terminus ribozyme, as predicted by Mfold, with the guide strand boxed shaded in gray. The Ago2 cleavage site is indicated by a black triangle (bp 10–11). The 5′ end nucleotide of AgoshRNA constructs and the base-pairing partner were replaced by A C (black circle). Hepatitis delta virus (HDV) and cytoplasmic polyadenylation element-binding protein (CPEB3) ribozymes (shown in blue) were inserted immediately downstream of the CUU 3′ terminus. The variable position 36 in the CPEB3 ribozyme is circled in blue. The ribozyme cleavage site is indicated by a star.

We previously designed and tested 21 anti-HIV AgoshRNAs. All HIV target sequences selected are highly conserved among virus isolates and subtypes. Seven of the 21 designed AgoshRNAs (Gag4-6, Pol1, Pol8, Pol45, and R/T5) were selected for this study because they showed significant anti-HIV activity in the absence of cellular toxicity. Figure 1A depicts the HIV genome with all target sites. This set includes three AgoshRNAs against overlapping Gag sequences starting at position 1364 (Figure 1B; AgoshGag4-6). Other AgoshRNAs were designed against important domains in the pol gene (encoding the viral Protease and Integrase enzymes) and the overlapping tat/rev genes. Figure 1C depicts the secondary RNA structure of a designed AgoshRNA molecule (AgoshR/T5) as predicted by Mfold. The anti-HIV guide is located on the 5′ side and is marked by a gray box. The predicted Ago2 cleavage site on the 3′ side of the duplex is indicated. We replaced the bottom base pair in these AgoshRNA molecules with an unpaired A C (circled) for optimal AgoshRNA activity.^32^ A self-cleaving ribozyme was inserted precisely at the 3′ terminus of the transcribed AgoshRNA downstream of the CUU overhang (Figure 1C). We selected the hepatitis delta virus (HDV) ribozyme and a ribozyme present in the mRNA encoding cytoplasmic polyadenylation element-binding protein 3 (CPEB3), because they do not require a specific sequence upstream of the cleavage site. Thus, these ribozymes could theoretically be used in combination with any AgoshRNA construct. The CPEB3 ribozyme is structurally related to the HDV ribozyme, but is significantly smaller.^41^ The CPEB3 ribozyme comes in two forms because of a SNP at position 36 (circled), which results in a G-C bp or a G⋅U wobble bp. We selected the G-C variant as it self-cleaves three times faster than the G⋅U variant.^41^ We inserted these two ribozymes individually at the 3′ terminus of the six anti-HIV AgoshRNA molecules. Theoretically, both ribozymes mediate self-cleavage exactly at the border of the AgoshRNA-ribozyme fusion, thus releasing the AgoshRNA molecule with an AgoshRNA-encoded 3′ CUU overhang (Figure 1C).

### Antiviral Activity of the AgoshRNA Inhibitors in Transient Assays

**Figure 2:**
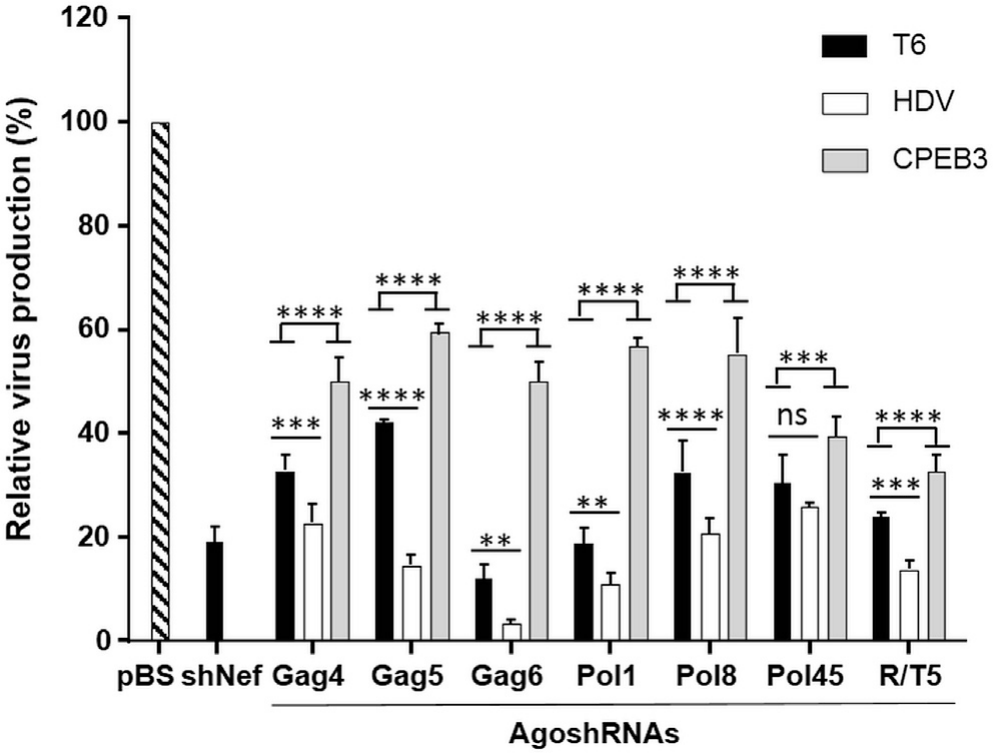
Inhibition of HIV Production by AgoshRNA Constructs HEK293T cells were co-transfected with 250 ng of the HIV pLAI, 1 ng of *Renilla* luciferase plasmid (pRL) and 25 ng of the shRNA/AgoshRNA constructs. Two days after transfection, inhibition of HIV production was determined by measuring CA-p24 levels in the culture supernatant. CA-p24 values were normalized to the *Renilla* luciferase activities. The ratio between the CA-p24 level and the *Renilla* luciferase activity in the presence of 25 ng pBS control was set at 100%. Bars represent the average values from six independent transfections, and error bars show the SD. Statistical analyses (two-way ANOVA followed by Tukey’s post hoc test) were performed, and differences among groups were considered significant when the corresponding p was < 0.05 (ns: not significant, p > 0.05; **p ≤ 0.01, ***p ≤ 0.001, and ****p ≤ 0.0001).

To evaluate whether insertion of a 3′ ribozyme improves the activity of AgoshRNA molecules, we first measured the antiviral activity of these constructs in transient transfection assays. Three AgoshRNA expression vectors were compared: the original construct with the T-stretch (T6-AgoshRNA) and the new designs with either the HDV or CPEB3 ribozyme (AgoshRNA-HDV and AgoshRNA-CPEB3, respectively). Each AgoshRNA expression construct (25 ng) was co-transfected in HEK293T cells with the HIV molecular clone pLAI (250 ng). A fixed amount of *Renilla* luciferase plasmid (1 ng) was included to control for variation in the transfection efficiency. HIV production was measured as the CA-p24 level in the culture supernatant at two days after transfection. CA-p24 levels were corrected for *Renilla* luciferase activity to calculate the relative level of virus production (Figure 2). Virus production in the presence of 25 ng pBluescript (pBS) control plasmid was set at 100%. A regular shRNA (shNef) served as the control for inhibition of virus production. AgoshRNA-T6 variants showed significant inhibitory activity with virus production levels dropping to between 18% and 42% versus the pBS control (p ≤ 0.0001). AgoshRNA-HDV variants consistently mediated more robust inhibition, with virus production levels dropping to values between 3% and 26% versus the pBS control (p ≤ 0.01), indicating a fold increase in activity compared with those of the matching AgoshRNA-T6 variants that range from 1.3- to 3.7-fold (Pol45 < Pol8 < Gag4 < Pol1 < R/T5 < Gag5 < Gag6). The inhibitory potency was significantly greater for the AgoshRNA-HDV variants than for the original AgoshRNA-T6 variants (p ≤ 0.01), with Pol45 as the exception. Surprisingly, the AgoshRNA-CPEB3 variants showed significantly reduced inhibitory activity compared with the AgoshRNA-T6 variants (p ≤ 0.001).

**Figure 3:**
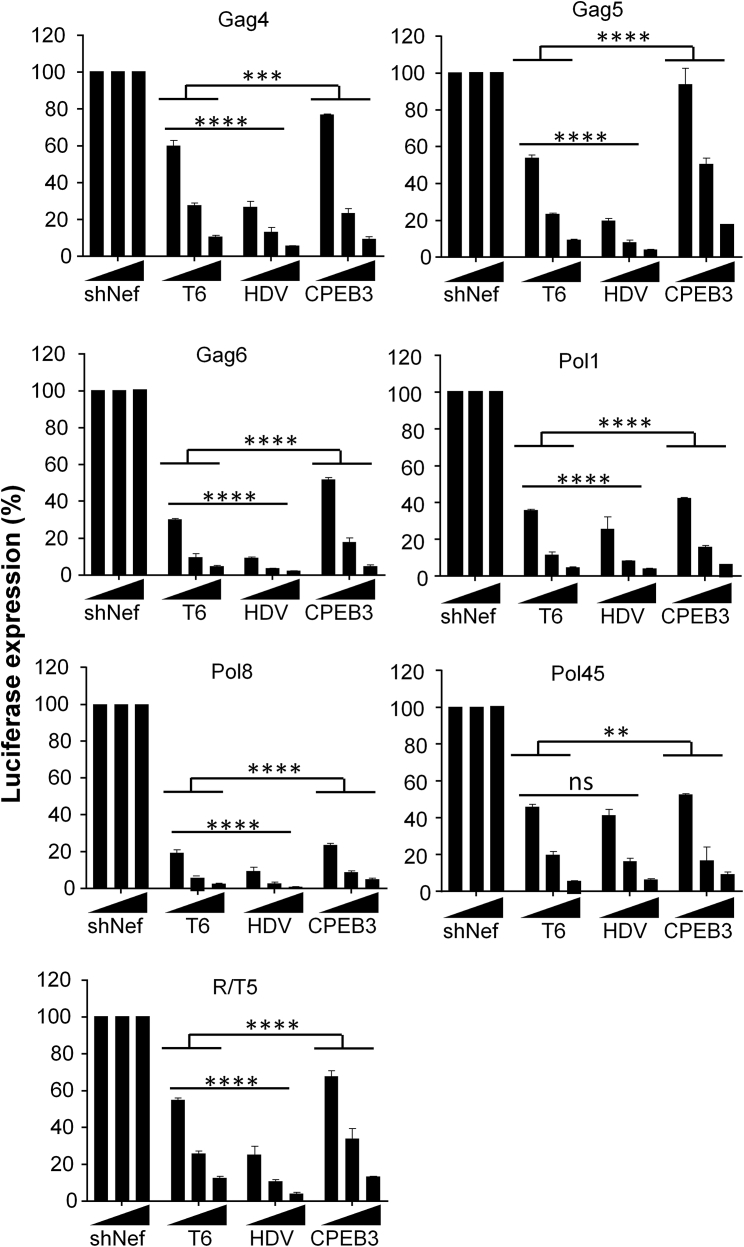
Luciferase Knockdown by AgoshRNAs Luciferase knockdown was determined by co-transfection of the reporters with the AgoshRNA constructs. HEK293T cells were co-transfected with 100 ng of the respective firefly luciferase reporter plasmid, 1 ng of *Renilla* luciferase plasmid, and an increasing amount of the AgoshRNA constructs (1, 5, and 25 ng). An irrelevant shRNA (shNef) served as the negative control, for which the activity was set at 100% luciferase expression. The mean values and SD are based on six independent transfections. Statistical analysis (two-way ANOVA) indicated that luciferase expression in the presence of antiviral AgoshRNAs differed significantly from luciferase expression measured with the shNef control (p ≤ 0.0001). AgoshRNA-HDV variants mediated significantly more robust inhibition than the AgoshRNA-T6 variant, whereas AgopshRNA-CPEB3 variants showed less inhibitory potency than the AgoshRNA-T6 variant (ns: no significant p > 0.05; **p ≤ 0.01, ***p ≤ 0.001, and ****p ≤ 0.0001).

We next tested the dosage effect of the differentially terminated AgoshRNA constructs in the luciferase reporter assay. HEK293T cells were co-transfected with an increasing amount of the AgoshRNA constructs (1, 5, and 25 ng), a fixed amount of the luciferase reporter construct (100 ng), and the *Renilla* luciferase control plasmid (1 ng). Two days after transfection, the relative luciferase expression was measured in cell lysates (Figure 3). The ratio between the luciferase and *Renilla* activity measured for the non-related shNef control plasmid was set at 100%. All AgoshRNA molecules showed significant inhibitory activity compared with the negative shNef control and suppression occurred in a dose-dependent manner (p ≤ 0.0001). The inhibitory potency was significantly greater for the AgoshRNA-HDV variants versus the original AgoshRNA-T6 variants (2- to 3-fold; p ≤ 0.0001), with Pol45 as a notable exception. The inhibitory potency was significantly reduced for AgoshRNA-CPEB3 versus AgoshRNA-T6 variants (p ≤ 0.0001–p ≤ 0.01), consistent with the results presented in Figure 2.

### Intracellular Processing of the AgoshRNA Transcripts

**Figure 4:**
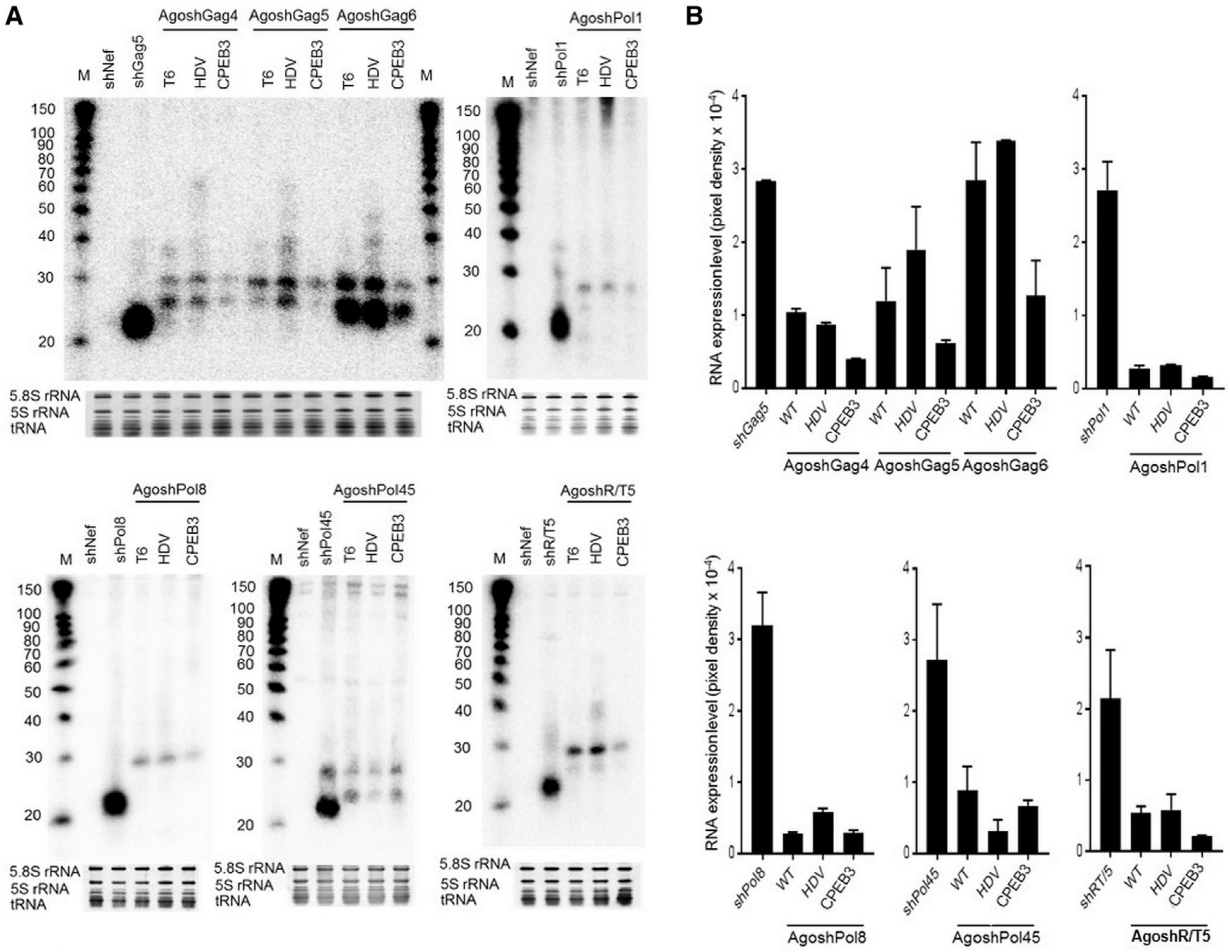
Northern Blotting of the AgoshRNA Processing Products (A) Processing of the AgoshRNA variants and the corresponding shRNA set was analyzed by Northern blot with a complementary probe. Five probes were designed to analyze the intracellular processing of the overlapping AgoshRNA triplet (AgoshGag4-6), AgoshPol1, AgoshPol8, AgoshPol45, and AgoshR/T5. Ethidium bromide staining of small rRNAs and tRNAs are shown as loading controls below the blot. (B) The RNA expression level was determined by quantitation of AgoshRNA products and 5S rRNA signal in (A). The AgoshRNA/5S ratio is plotted in pixel density. Similar results were observed in at least two independent transfection experiments.

The range of silencing activities observed for the differentially terminated AgoshRNA molecules may reflect quantitative or qualitative differences in RNA processing by Ago2 or differences in intracellular stability of the transcripts. We performed Northern blot analysis to examine AgoshRNA expression. The same molar amount of the AgoshRNA constructs was transfected into HEK293T cells. Total cellular RNA was extracted two days after transfection, and a fixed amount (5 μg) was subjected to Northern blotting with a 5′ side probe. Five probes were designed to analyze the intracellular processing of the overlapping AgoshRNA triplet (AgoshGag4-6) and the other AgoshRNAs (Figure 4A). We compared the T6, HDV, and CPEB3 constructs for the complete AgoshRNAs set. The non-related shNef served as the negative control, and regular shRNAs complementary to the different probes (shGag5, shPol1, shPol8, shPol45, and shR/T5) were included as the positive control. Each probe would detect the ∼30 nt guide strand and the trimmed products (∼24 nt fragments) for the AgoshRNA constructs and the ∼21 nt fragment for regular shRNAs.

Two RNA products were observed for all variants (T6, HDV, and CPEB3) of the AgoshRNA triplet (AgoshGag4-6), AgoshPol45, and AgoshR/T5, the ∼30 nt guide strand generated by Ago2 cleavage and the PARN-trimmed products of ∼24 nt (Figure 4A). Only a single band was observed for AgoshPol8 (∼30 nt) and AgoshPol1 (∼27 nt), which may suggest that only the Ago2-cleaved product or the trimmed product is produced. *In vitro* and *in vivo* experiments have shown that 3′ trimming of Ago2-cleaved pre-miRNAs is not essential for silencing activity and that the RISC is functional with guide RNAs of different lengths.^18^ Preferential accumulation of certain intermediates was associated with a PARN preference for specific nucleotides, and it was suggested that the trimming reaction prefers adenosine to uridine.^18^ Thus, the nucleotide composition of the AgoshRNA stem could explain the differential accumulation of certain intermediates. However, we did not observe a clear trend for the different AgoshRNA molecules. The inserted ribozymes efficiently cleaved the precursor transcript of 132 nt (HDV) and 114 nt (CPEB3) to generate the precise AgoshRNA duplex of 42 nt with a discrete 3′ overhang that was subsequently cleaved (∼30 nt) and trimmed (∼24 nt) by Ago2 and PARN, respectively (Figure 4A). The addition of an HDV or CPEB3 ribozyme at the 3′ terminus of the AgoshRNA sequence did not change the normal Ago2 cleavage and PARN trimming as the same fragment sizes were observed for the ribozyme variants compared with the original AgoshRNA-T6 constructs.

Prominent ∼21 nt bands were generated by all Dicer-processed control shRNAs (Figure 4A). Fewer RNA signals were produced by most AgoshRNA constructs than the original shRNAs, consistent with previous studies.^25^, ^37^ The AgoshGag6 product was the exception, as it was more abundant than the other AgoshRNAs, consistent with its increased activity (Figures 2 and 3). Quantitation of the RNA signals indicates that HDV ribozyme insertion can increase the amount of RNA product (Figure 4B). For instance, insertion of the HDV ribozyme in AgoshGag5, AgoshGag6, and AgoshPol8 increased the amount of AgoshRNA-processed products, consistent with the observed superior inhibitory activity (Figures 2 and 3). However, this gain was not apparent for AgoshGag4, AgoshPol1, and AgoshR/T5, although enhanced inhibition was observed for most of these AgoshRNA-HDV constructs (Figures 2 and 3). These results may cautiously suggest that a precise 3′ end can boost the silencing activity of AgoshRNAs. Insertion of the HDV ribozyme in AgoshPol45 resulted in a reduced amount of processed AgoshRNAs but a similar silencing activity was measured confirming the notion of increased silencing activity. Thus, HDV ribozyme insertion can benefit the AgoshRNA design at two levels: increased expression and increased activity. Northern blot analysis demonstrated that insertion of the CPEB3 ribozyme resulted in a greatly reduced level of both Ago2-cleaved and PARN-trimmed products, consistent with the reduced activity that was measured.

### Antiviral Activity in Stably Transduced T Cells and HIV Escape Options

**Figure 5:**
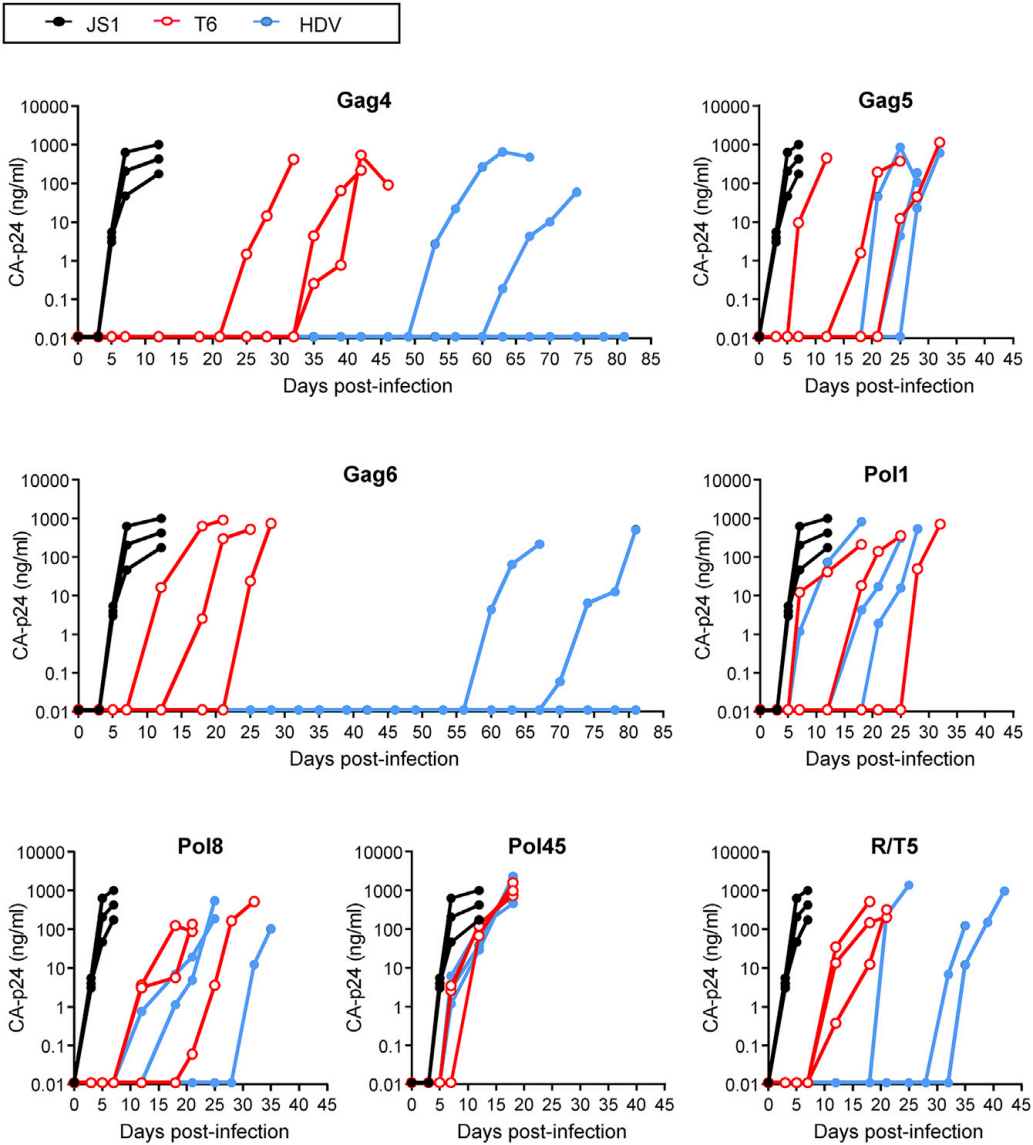
Kinetics of HIV LAI Replication in AgoshRNA-Expressing Cells Three parallel infections per AgoshRNA-expressing SupT1 cell line were performed. The empty lentiviral vector JS1 served as the negative control in all experiments. CA-p24 antigen was measured starting at day 3 after infection up to day 81.

To test HIV inhibition in a spreading virus infection, we transduced the SupT1 T cell line with lentiviral constructs encoding the original AgoshRNA-T6 and the new AgoshRNA-HDV inhibitors at a MOI of 0.15. Cells were subjected to fluorescence-activated cell sorting (FACS) to determine GFP expression after 3 days and subsequently challenged with the X4-tropic primary HIV isolate LAI at a MOI of 0.1. Infections were prolonged to monitor virus evolution and performed in triplicate because evolution is a chance process. Cells transduced with the empty JS1 vector were also GFP sorted and served as the negative control. Viral CA-p24 production was monitored starting at day 3 after infection up to day 81. All infections were performed in parallel, but were grouped according to the viral target in seven graphs (Figure 5). The JS1 negative control was included in each graph.

**Figure 6:**
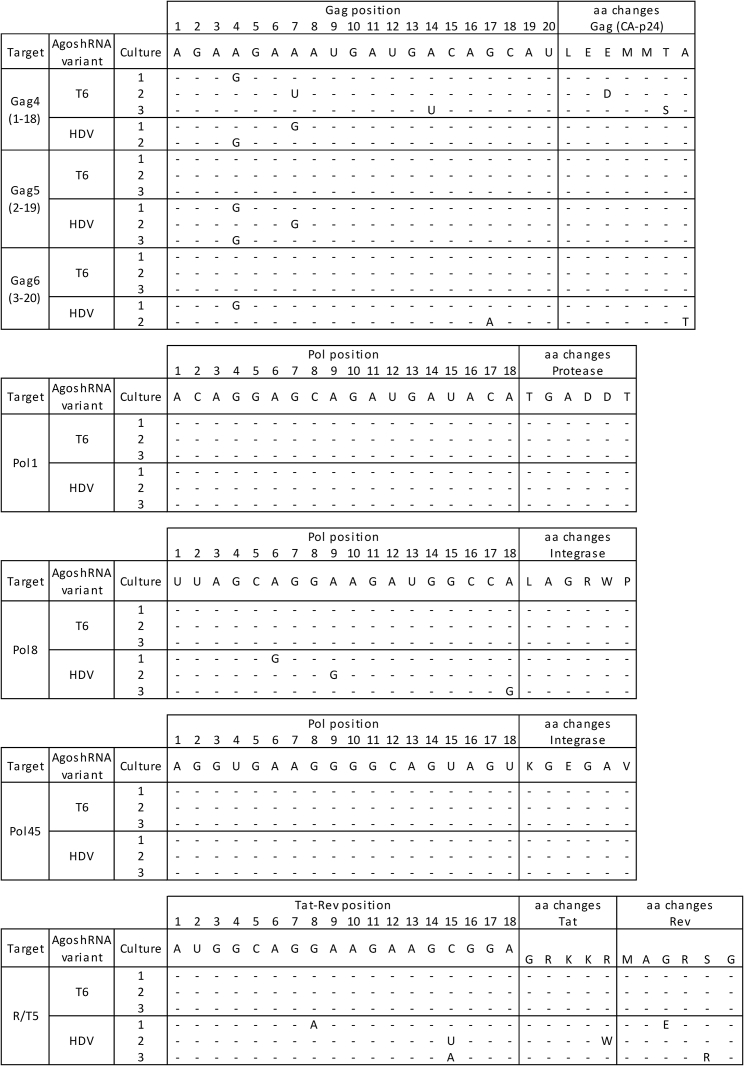
HIV Sequence Variation upon AgoshRNA Escape Clonal sequence analysis of the target sequences upon viral escape on the AgoshRNA-expressing SupT1 T cell line. Changes in the sequence compared with wild type are indicated. The targets are shown from 5′ to 3′; position 1–20 for overlapping Gag sequences and 1–18 for Protease, Integrase, and Tat-Rev sequences. Amino acid changes are shown in the right column.

Virus replication was delayed in all AgoshRNA-expressing cells compared with the control JS1 cells, with differential inhibitory activity among the AgoshRNA constructs. We will first describe the results for AgoshGag4. It took around 25–35 days for viral escape to occur in all three T6-cultures (Figure 5, upper left panel). Virus replication was much delayed or even prevented in the HDV cultures. Two HDV cultures yielded a CA-p24-positive supernatant around days 53–64 and no CA-p24 was measured for the third culture up to day 81. We screened for the selection of truly AgoshRNA-resistant HIV variants by passage of cell-free virus on AgoshRNA-expressing SupT1 cells and control nontransduced SupT1 cells. For all five escape cultures, the passaged virus replicated equally well on these cells (data not shown), confirming that an AgoshRNA-resistant virus variant was selected. Next, the 18 nt HIV targets and flanking regions were analyzed by population-based sequencing. A single point mutation was detected in the AgoshGag4 target sequence of all five escape cultures, confirming the selection pressure imposed by the AgoshGag4 inhibitor (Figure 6). Since all targets were selected as conserved HIV genome regions that encode essential viral proteins, mutations within the target may affect the encoded amino acid (non-silent), although silent codon changes are also possible. We listed the amino acid changes in the last column of Figure 6.

Similarly, AgoshGag5-T6 challenged cultures yielded a CA-p24-positive supernatant quite early around days 7–25, whereas HDV cultures showed a moderately delayed peak of virus replication around days 18–28 (Figure 5, upper right panel). The phenotype test indicated that T6-escape viruses did not replicate on restricted cells, whereas HDV escape viruses replicated well on restricted cells and indeed point mutations in the target sequence were detected only for the HDV set (Figure 6). It seems that no resistant virus was selected in AgoshRNA-T6 expressing cells, likely because no sufficient selective pressure was put on HIV. AgoshGag6-T6 challenged cultures yielded a CA-p24-positive supernatant around days 7–25, but virus replication was strikingly delayed in two HDV cultures (peak infection around days 60–70) and blocked in a single culture. The phenotype test indicated that only HDV escape viruses replicated well on restricted cells and true escape was demonstrated by the acquisition of point mutations within the 18 nt HIV target (Figure 6). The same phenomenon was observed for AgoshPol8 and AgoshR/T5. Virus replication was delayed in HDV variant cultures compared with T6 variant cultures, with differential inhibitory activity among the AgoshRNA constructs. Point mutations within the 18 nt HIV target were exclusively detected for HDV cultures (Figure 6). The phenotype test indicated that the candidate AgoshPol1 and AgoshPol45 escape viruses (both T6 and HDV variants) did not replicate on restricted cells, indicating viral breakthrough replication because of poor inhibition, which also explains the absence of resistant mutations.

There is a preference for silent codon changes, reflecting the selection pressure on the virus to maintain functionally active proteins. For instance, we scored seven silent codon changes and only three non-silent changes in Gag (CA-p24) and all codon changes in Integrase were silent. The Tat-Rev overlap provides a special situation since double silent codon changes are nearly impossible because of the overlapping reading frames. Two mutants are silent in Tat and only one in Rev. The targeted HIV sequence encodes critical Tat amino acids, but a less important Rev domain. The Tat target encodes a basic stretch of amino acids (48GRKKR52) in the first coding exon of Tat and Rev that encodes the nuclear localization signal and the TAR-binding domain.^42^ In fact, the amino acid change found in Tat (R52W) represents a natural sequence variation, indicating that the virus is under pressure to maintain the Tat function during AgoshRNA escape.^43^ The escape data indicate that the virus does not use all 18 positions within the target to become resistant to AgoshRNA. For the largest Gag4-6 dataset, it is remarkable that mutations do not cluster in the central region. Inspection of the codons may provide a simple explanation, as there are two adjacent AUG codons for methionine, which cannot be mutated in a silent manner. In general, mutations are dominated by transitions (n =13) over transversions (n = 3), consistent with previous HIV evolution studies.^44^, ^45^

### Evaluation of AgoshRNA Toxicity in a Human T Cell Line

We next evaluated whether the insertion of an HDV ribozyme at the 3′ terminus of the transcript can induce adverse effects. We transduced SupT1 cells with the respective LV and the empty JS1 vector served as negative control. To determine any negative effects on the physiology and growth of transduced cells, the percentage of GFP-positive, transgene-expressing cells in the culture was monitored for 81 days (Figure 5). Nontransduced cells formed the internal control in these mixed cultures. The transduction was performed at the high MOI of 1.5. We included the antiviral molecule shGag5, which was previously shown to trigger reduced cell growth *in vitro* and *in vivo*.^46^ We confirmed the selective loss of GFP-positive cells for shGag5, but none of the AgoshRNA-T6 and AgoshRNA-HDV cultures showed such cell growth impairment (Table 1). A significant decrease in the percentage of GFP-positive cells was observed exclusively for shGag5 versus the JS1 control (p ≤ 0.0001) and the AgoshRNA (p ≤ 0.001).

**Table 1:** Competitive Cell Growth Assay

Lentiviral Vector Construct	Change in Proportion of GFP^+^ Cells (%)			
	T6		HDV	
	Mean	SD	Mean	SD
Empty JS1	2.3	0.5	—	—
AgoshGag4	4.8	2.4	4.0	1.2
AgoshGag5	1.5	5.2	3.3	2.6
AgoshGag6	0.1	2.5	−0.7	1.2
AgoshPol1	−1.5	1.8	−0.8	0.9
AgoshPol8	−1.4	3.1	−2.5	1.6
AgoshPol45	4.9	1.3	5.3	0.7
AgoshR/T5	2.1	0.8	1.8	0.4
Toxic shGag5	22.9	0.5	—	—

## Discussion

We previously defined several parameters for the design of optimized AgoshRNA molecules: a small 5 nt loop and a duplex length of 18 bp with a bottom A C mismatch.^31^, ^32^, ^34^ We successfully designed 21 AgoshRNAs that were active in reporter silencing, but only two of these exhibited profound HIV inhibition in spreading virus infections in a T cell line. Selection of AgoshRNA-resistant virus variants could be demonstrated for only one of these antivirals, arguing that the other did not exert sufficient selective pressure on the HIV RNA genome.^39^, ^44^ Therefore, we argue that further refinement of the AgoshRNA design is essential to increase the silencing activity.

In this study, we focused on optimization of the 3′ end of these AgoshRNA molecules. Pol III promoters such as 7SK, U6, and H1 are widely used for the expression of small transcripts, such as shRNA and AgoshRNA molecules. These promoters use a precise transcription start site, and transcription is terminated at a T-stretch. The termination site within the T-stretch is quite heterogeneous, and consequently small RNAs have a variable U-tail of 1–6 nt.^40^ It has been suggested that Ago2 binding is dependent on the length of the 3′ overhang, which is directly influenced by the transcriptional termination site. For instance, synthetic AgoshRNAs with a 3′ overhang up to 3 nt are more potent than overhangs with more than 3 nt.^24^, ^25^ We inserted a self-cleavage ribozyme (HDV or CPEB3) immediately downstream of the AgoshRNA sequence to create a precise 3 nt 3′ overhang in the context of the duplex (Figure 1C). The inserted ribozyme efficiently self-cleaved the transcribed RNA and did not cause differential AgoshRNA processing compared with AgoshRNAs with a regular T6 termination signal (Figure 4). Introduction of these two ribozymes had an opposite effect on the inhibitory capacity of the AgoshRNA antivirals. Insertion of the HDV ribozyme resulted in superior silencing activity, whereas insertion of the CPEB3 ribozyme resulted in a loss of inhibitory activity.

These two ribozymes also had a differential effect on RNA production. Northern blot analysis demonstrated that several AgoshRNA-HDV produced more RNA product, whereas insertion of the CPEB3 ribozyme resulted in less RNA product (Figure 4B). The reduced concentration of AgoshRNA-CPEB3 is most likely linked to the reduced cleavage rate of the CPEB3 ribozyme compared with the HDV ribozyme,^47^ although no precursor transcripts were ever detected in our studies. The AgoshRNA-CPEB3 transcripts may exhibit reduced stability, possibly because of the reduced self-cleavage activity of the CPEB3 ribozyme compared with the HDV ribozyme.

On the other hand, insertion of the HDV ribozyme significantly increased the antiviral activity of all AgoshRNA molecules, independent of the RNA abundance. This indicates that the abundance of AgoshRNA molecules is not the only determinant of AgoshRNA activity. The length of the 3′ end overhang of the duplex may also contribute to the silencing activity, possibly through a structural effect on Ago2-mediated processing and silencing, as previously suggested.^24^, ^25^ The shorter 3′ overhang seems to be preferred by Ago2. Thus, removal of the U-tail may facilitate increased interaction with Ago2 to improve the silencing activity. The selective pressure exerted by these optimized AgoshRNA-HDV molecules led to the selection of resistant virus variants, thus confirming their antiviral potency. Furthermore, the AgoshRNA-HDV molecules revealed no toxicity in the ultra-sensitive competitive cell growth assay. Taken together, these results indicate that the AgoshRNA-HDV design is optimal for the future development of therapeutics. The AgoshRNA design has a major hypothetical advantage that only a single active guide RNA is generated, preventing unwanted off-targets effects that can be induced by the passenger strand of regular shRNAs. In addition, Dicer-independent shRNAs is likely to be the silencing method of choice for certain cell types, e.g., monocytes that lack Dicer.^48^ We previously described additional advantages of the AgoshRNA design.^26^, ^29^

## Materials and Methods

### Plasmid Construction

For the AgoshRNA and shRNA constructs, complementary DNA oligonucleotides encoding the corresponding sequences were annealed to create sticky *Bam*HI and *Hin*dIII sites and subsequently were inserted into the corresponding restriction sites of the pSUPER vector.^1^ The vector uses the human Pol III H1 promoter to express the AgoshRNA transcript. Firefly luciferase reporter constructs (pGL3; Promega, Madison, WI, USA) were made by insertion of a 50–70 nt HIV sequence, with the 18 nt target region in the center, in the *Eco*RI and *Pst*I sites of the pGL3 plasmid.^49^ The precise positions of the inserted HIV fragments are as follows: Gag (1341–1399), Pol1 (1889–1948), Pol8 (4017–4155), Pol45 (4531–4589), and R/T5 (5535–5583). The luciferase reporters with the target sequences were described previously.^22^ All constructs were sequence-verified using the BigDye Terminator Cycle Sequencing kit (Applied Biosystems, Foster City, CA, USA). For sequencing of these constructs a sample denaturation temperature of 98°C was used, and 1 M betaine was included in the reaction mixture.

### Cell Culture

HEK293T adherent cells (ATCC CRL-11268) were grown as a monolayer in DMEM (Invitrogen, Life Technologies, Carlsbad, CA, USA) supplemented with 10% fetal calf serum (FCS), penicillin (100 U/mL), streptomycin (100 μg/mL), and minimal essential medium non-essential amino acids (DMEM/10% FCS) in a humidified chamber at 37°C and 5% CO_2_. SupT1 T cells (ATCC CRL-1942) were grown in Advanced RPMI (Gibco BRL, Carlsbad, CA, USA) supplemented with l-glutamine, 1% FCS, penicillin (30 U/mL), and streptomycin (30 μg/mL) in a humidified chamber at 37°C and 5% CO_2_. Regular testing for mycoplasma contamination was performed.

### Transient HIV Inhibition Assay

To determine inhibition of virus production, HEK293T cells were seeded 1 day before transfection in 24-well plates at a density of 1.2 × 10^5^ cells/well in 500 μL DMEM/10% FCS without antibiotics. The cells were co-transfected using Lipofectamine 2000 with 250 ng of the full-length HIV molecular clone pLAI,^50^ 1 ng of pRL-CMV and 25 ng of pSuper-AgoshRNA construct. We added pBS to ensure equal DNA concentration per transfection. A known anti-HIV shRNA (shNef) served as a positive control for virus inhibition.^49^ Two days after transfection, virus production was determined by measuring CA-p24 levels in the culture supernatant by ELISA. Cell lysates were made to measure *Renilla* luciferase activity with the *Renilla* Luciferase Assay System (Promega, Madison, WI, USA), according to the manufacturer’s instructions. The relative CA-p24 production was calculated as the ratio between the CA-p24 level and the *Renilla* luciferase activity. We performed three independent transfections, each in duplicate. Values were corrected for between-session variation, as described previously.^51^

### Luciferase Assays

For luciferase assays, HEK293T cells were seeded 1 day before transfection in 24-well plates at a density of 1.2 × 10^5^ cells/well in 500 μL DMEM/10% FCS without antibiotics. Cells were transfected with 100 ng of the firefly luciferase expression plasmid; 1 ng of the *Renilla* luciferase expression plasmid (pRL); and 1, 5, or 25 ng of pSuper-AgoshRNA vector, using the Lipofectamine 2000 reagent (Invitrogen) according to the manufacturer’s protocol. We added pBS plasmid to create an equal DNA concentration for each transfection. Cells were lysed 2 days after transfection to measure firefly and *Renilla* luciferase activities using the Dual-Luciferase Reporter Assay System (Promega, Madison, WI, USA). The relative luciferase activity was calculated as the ratio between firefly and *Renilla* luciferase activities and corrected for between-session variations. The pBS plasmid and an unrelated shRNA (shNef) served as negative controls. The luciferase activity scored with shNef activity was set at 100%. We performed three independent transfections, each in duplicate. The resulting six values were used to calculate the SD, shown as error bars.

### Northern Blot Analyses

For siRNA analyses, 1.5 × 10^6^ HEK293T cells were seeded in T25 flasks in 4 mL DMEM/10% FCS without antibiotics. The cells were transfected with equimolar amounts of AgoshRNA and shRNA constructs (equivalent to 1 μg of vector), using the Lipofectamine 2000 reagent. Small RNAs were isolated 2 days after transfection, using the mirVana miRNA isolation kit (Ambion, Life Technologies, Austin, TX, USA) according to the manufacturer’s protocol. RNA concentrations were determined on the Nanodrop 1000 (Thermo Fisher Scientific, Waltham, MA, USA). Five micrograms of total RNA was heated for 5 min at 95°C and then resolved in a 15% denaturing polyacrylamide gel (Precast Novex TBU gel; Life Technologies). The γ [^32^P]-labeled decade RNA marker (Life Technologies) was used for size estimation. To check for equal sample loading, the gel was stained in 2 μg/mL ethidium bromide for 20 min and visualized under UV light. The RNA in the gel was transferred to a positively charged nylon membrane (Boehringer Mannheim). Locked nucleic acid (LNA) oligonucleotides were 5′ end labeled with the kinaseMax kit (Ambion) in the presence of 1 μL γ [^32^P]-ATP (0.37 MBq/μL; PerkinElmer, Waltham, MA, USA). Sephadex G-25 spin columns (GE Healthcare, Little Chalfont, UK) were used to remove the unincorporated nucleotides. We used the following oligonucleotides (LNA-positions are underlined): Gag4-6: 5′-GAAGAAATGATGACAGCAT-3′, Pol1: 5′-ACAGGAGCAGATGATACAG -3′, Pol8: 5′-TTAGCAGGAAGATGGCCAGT-3′, Pol45: 5′-GTGAAGGGGCAGTAGTAAT-3′ and R/T5: 5′-ATGGCAGGAAGAAGCGGAG-3′. The membrane was incubated with labeled LNA oligonucleotides in 10 mL ULTRAhyb hybridization buffer overnight at 42°C. The membrane was washed twice for 5 min at 42°C with 2× saline sodium citrate (SSC)/0.1% SDS and twice for 5 min at 42°C with 0.1× SSC/0.1% SDS. The signals were captured by Typhoon FLA 9500 (GE Healthcare) and quantitated using ImageJ software.

### Lentiviral Vector Production and Transduction

The expression cassette for the anti-HIV AgoshRNA was cloned in the third-generation, self-inactivating LV JS1 (pRRLcpptpgkgfppreSsin).^52^ The AgoshRNA cassette was excised with *Pst*I/*Xho*I and inserted in the multiple cloning site (*Pst*I/*Xho*I) of JS1, resulting in JS1-AgoshRNA. This vector expresses an anti-HIV AgoshRNA from the human Pol III H1 promoter and the GFP reporter from the human polymerase-II PGK promoter. The LV was produced and titrated, as described previously.^39^ The vector was produced by co-transfection of LV plasmid and packaging plasmids pSYNGP^53^, pRSV-rev, and pVSV-g^54^ with Lipofectamine 2000 (Invitrogen, Life Technologies). After transfection, the medium was replaced with OptiMEM (Invitrogen, Life Technologies). The LV-containing supernatant was collected and filtered (0.45 μm), and aliquots were stored at −80°C. The transduction titer was measured via GFP expression. SupT1 cells were transduced at a MOI of 0.15. Three days after transduction, live cells were selected by FACS for GFP expression.

### HIV Infection

The HIV LAI stock was produced by transfection of HEK293T cells with the pLAI molecular clone. Cell-free viral stocks were passed through a 0.2 μm pore size filter and titrated on SupT1 cells, measuring virus production by CA-p24 ELISA. SupT1 (3 mL cultures in 6-well plates, 1 × 10^6^ cells/well) were challenged with HIV LAI at a MOI of 0.1. Virus spread was monitored by scoring of syncytia formation every two days and by measuring CA-p24 production. Cells were passaged twice a week.

### Sequencing Proviral Target Regions

When virus replication was observed after infection with HIV LAI, cellular DNA with the integrated proviruses was isolated as previously described.^55^ Integrated proviral DNA sequences were PCR amplified with the following primer pairs (the position within pLAI is indicated): Gag sense (5′-CAGACCATCAATGAGGAAGCTGCAGAATGGGAT-3′, position 1445) and antisense (5′-CCCTGGCCTTCCCTTGTAGGAAAACCAGATCTTCCC-3′, position 2141); Protease, sense (5′-GTCAGAGCAGACCAGAGCCAACAG-3′, position 2183) and antisense (5′-GATATTTCTCATGTTCATCTTGGGCCTTATCTATTCC-3′, position 2659); Integrase, sense (5′-GGCAACTAGATTGTACACATTTAGAAGG-3′, position 4499) and antisense (5′-CTCTTTTTCCTCCATTCTATGGAGA-3′, position 5377); and Tat-Rev sense (5′-ATATCAAGCAGGACATAACAAGG-3′, position 5525) and antisense (5′-TGCTTTAGCATCTGATGCACAAAATA-3′, position 6458) with 30 cycles (1 min denaturation at 96°C, 1 min annealing at 62°C, and 2 min extension at 72°C). The PCR products were sequenced with the Big Dye Terminator Cycle Sequencing kit (Applied Biosystems, Foster City, CA) using one of the indicated primers.

### Competitive Cell Growth Assay

Lentivirus transduction of SupT1 T cells was performed with a MOI of 1.5. Transduced SupT1 T cells were screened for a negative impact on cell growth (induced by lentiviral integration and/or AgoshRNA expression) in the CCG assay.^56^ In brief, the mixture of transduced (GFP^+^/AgoshRNA^+^) and nontransduced (GFP^−^) cells was monitored for up to 81 days for the GFP^+/−^ ratio by FACS. The impact on cell growth was measured as change in proportion of GFP^+^ cells (%).

### Statistical Analysis

Results are presented as means ± SD. p < 0.05 was considered statistically significant. Comparisons between three or more groups were analyzed by two-way ANOVA followed by Tukey’s post hoc test. Statistical analysis was performed using GraphPad Prism 7.02 (GraphPad, La Jolla, CA, USA).

## Author Contributions

E.H.-C. designed and conducted the experiments. Z.G. performed the Northern blot analyses. E.H.-C. and B.B. drafted the manuscript. E.H.-C. and B.B. analyzed the data.

## Conflicts of Interest

The authors declare no competing interests.
